# Automatic Separation of Respiratory Flow from Motion in Thermal Videos for Infant Apnea Detection

**DOI:** 10.3390/s21186306

**Published:** 2021-09-21

**Authors:** Ilde Lorato, Sander Stuijk, Mohammed Meftah, Deedee Kommers, Peter Andriessen, Carola van Pul, Gerard de Haan

**Affiliations:** 1 Department of Electrical Engineering, Eindhoven University of Technology, 5612 AZ Eindhoven, The Netherlands; s.stuijk@tue.nl (S.S.); G.d.Haan@tue.nl (G.d.H.); 2 Department of Family Care Solutions, Philips Research, 5656 AE Eindhoven, The Netherlands; mohammed.meftah@philips.com; 3 Department of Neonatology, Máxima Medical Centre, 5504 DB Veldhoven, The Netherlands; Deedee.Kommers@mmc.nl (D.K.); P.Andriessen@mmc.nl (P.A.); 4 Department of Applied Physics, Eindhoven University of Technology, 5612 AZ Eindhoven, The Netherlands; C.vanPul@mmc.nl; 5 Department of Clinical Physics, Máxima Medical Centre, 5504 DB Veldhoven, The Netherlands

**Keywords:** thermal camera, respiration, apnea, respiratory flow, thermography, obstructive apnea, unobtrusive, vital signs, infant, neonatal, NICU

## Abstract

Both Respiratory Flow (RF) and Respiratory Motion (RM) are visible in thermal recordings of infants. Monitoring these two signals usually requires landmark detection for the selection of a region of interest. Other approaches combine respiratory signals coming from both RF and RM, obtaining a Mixed Respiratory (MR) signal. The detection and classification of apneas, particularly common in preterm infants with low birth weight, would benefit from monitoring both RF and RM, or MR, signals. Therefore, we propose in this work an automatic RF pixel detector not based on facial/body landmarks. The method is based on the property of RF pixels in thermal videos, which are in areas with a smooth circular gradient. We defined 5 features combined with the use of a bank of Gabor filters that together allow selection of the RF pixels. The algorithm was tested on thermal recordings of 9 infants amounting to a total of 132 min acquired in a neonatal ward. On average the percentage of correctly identified RF pixels was 84%. Obstructive Apneas (OAs) were simulated as a proof of concept to prove the advantage in monitoring the RF signal compared to the MR signal. The sensitivity in the simulated OA detection improved for the RF signal reaching 73% against the 23% of the MR signal. Overall, the method yielded promising results, although the positioning and number of cameras used could be further optimized for optimal RF visibility.

## 1. Introduction

Respiration is one of the most important vital signs, able to detect early clinical decline [1]. It can be monitored in hospital wards to detect critical events and respiratory irregularities. In Neonatal Intensive Care Units (NICUs), in particular, the immaturity of the respiratory control system of premature infants is the main cause of Apnea Of Prematurity (AOP), which is one of the most common diagnoses [2]. Infants’ breathing patterns can present Cessations of Breathing (COBs), the ones that last 20 s or 10 s accompanied by bradycardia and/or desaturation are called apneas [3]. Three main categories of apneas can be defined: Central Apnea (CA), which is characterized by cessation of both respiratory flow and effort, Obstructive Apnea (OA) which is caused by the collapse of the upper airway, and it manifests as cessation of airflow and presence of respiratory effort, and Mixed Apnea (MA) which is a mixture of the previous two [2].

Infants in NICUs are typically monitored using several adhesive skin sensors and electrodes. Respiration is monitored using Chest Impedance (CI), which is measured using ECG electrodes. However, this method is not able to detect OAs, due to the presence of respiratory effort [4,5]. Discriminating between different types of apnea is difficult due to limitations of the technology used. Moreover, both CI and other methods that may be used to improve the detection of apneas require attaching sensors and electrodes to the skin or positioning sensors close to the nostrils. This causes discomfort or even skin irritation in preterm infants. Because of these reasons, unobtrusive monitoring of vital signs, and in particular of respiration is being investigated.

Numerous technologies are being researched for the unobtrusive monitoring of respiration: radars [6,7], RGB and Near-Infrared (NIR) cameras [8,9], thermal cameras or infrared thermography [10,11,12], pressure-sensitive mattresses [13,14], and vision systems with depth sensing [15,16]. All these techniques, apart from thermal cameras, can monitor only respiratory motion. The clear advantage in monitoring both respiratory flow and motion is the more accurate detection and classification of apneas. Identifying the type of apnea is clinically relevant as the required therapeutic intervention can be different [5]. Studies suggest that the phase shift between abdomen motion and thorax motion can also be monitored using normal RGB/NIR cameras or thermal cameras and could be used to identify OAs [17,18]. However, the identification of the regions can be quite challenging, especially for infants or in general if the subject is covered by a blanket or sheet. Infrared thermography and analysis of both respiratory motion and flow remains the most promising option when aiming at unobtrusive apnea detection and classification.

Studies using thermal cameras for the detection of respiratory flow have been published in recent years. Several works proposed to detect respiratory flow based on a manually selected Region Of Interest (ROI) [10,18,19,20,21,22,23], others used facial and/or body landmarks based solely on thermal images [23,24,25,26] or by combining it with RGB/NIR images [27,28,29,30]. Moreover, works that propose to analyze separately respiratory flow and motion in thermal recordings have been already proposed [12,18,25,27]. However, these required manually selected ROIs, textiles positioned on the subject’s face to amplify respiratory flow, combination of thermal and non-thermal camera solutions, and/or facial/body landmark detection. Although facial and body landmark detection can be used in controlled settings, using them in hospital settings without any constraints on the subject’s position is quite challenging [31]. Moreover, the identification of multiple ROIs is required, considering that patients could be nose or mouth-breathing. In addition, by selecting ROIs specifically at the nose/mouth areas the thermal variations due to respiratory flow which can be registered in the environment, e.g., on the pillow, would be ignored. This is, sometimes, the only source of respiratory flow in the video, as will be shown in this publication. Recent solutions propose to automatically identify respiratory pixels in thermal videos [31,32,33]. These methods, however, have the disadvantage of mixing the thermal variations due to respiratory flow and motion into a single signal, obtaining a mixed respiratory signal (the terminology used is detailed in Section 2.1).

For these reasons, we propose in this work an automatic respiratory flow pixel detector using characteristics that set respiratory flow and respiratory motion pixels apart. In particular, respiratory flow pixels can be in non-edge areas of the videos, and due to the thermal diffusion, the airflow generates areas with a smooth gradient. Additionally, respiratory motion can be in phase or in anti-phase with the respiratory flow. The use of this algorithm results in a respiratory flow signal, which can be used for a more accurate detection and classification of apneas. The algorithm will be applied to a set of low-resolution infants’ thermal videos collected in a neonatal ward. The data amounts to a total of 132 min split between 9 infants. Finally, we adapted the videos to simulate OAs and used these as a proof of concept for apnea detection using a COB-detector. The performance in the apnea detection was analyzed for the different respiration signals obtainable from a thermal video. In particular, we compared the performance of the respiratory flow signal, obtained with the method proposed in this publication, with the one of a mixed respiratory signals which is obtained using the method explained in [33].

## 2. Materials and Methods

### 2.1. Background and Definitions

In thermal videos, respiration can be detected by monitoring Respiratory Flow (RF), Respiratory Motion (RM), and/or both. The videos, therefore, contain RM pixels, RF pixels, and noise-pixels. Pixels simultaneously affected by RF and RM can also be present, the term Mixed Respiratory (MR) pixels is used to indicate the pixels belonging to this category as well as the ones belonging to the RF or RM pixels. Therefore, we define:

- *RF signal*: the signal obtained by combining only the RF pixels.
- *RM signal*: the one obtained using only the RM pixels.
- *MR signal*: this signal can be obtained by combining respiration pixels, regardless their origin, i.e., the MR pixels. This is the signal that was obtained in earlier research [31,32,33].

Aiming at apnea detection and classification, we need to monitor the *RF signal* since this allows us to accurately detect OAs. However, to differentiate between an OA and a CA, motion information is also needed. It could be possible to monitor the *RM signal*, but the *MR signal* can also be used as it will become a *RM signal* in the windows containing apneas (since the flow contribution will not be present in those segments). Based on this reasoning we aim at monitoring the *RF signal* and the *MR signal* which can potentially ensure an accurate apnea classification and detection.

To obtain the *RF signal*, a preferably automatic selection of the RF pixels is needed. To arrive at an automatic detection, we may use the following characteristics:

1. RM pixels are located at (typically sharp) edges in the thermal image, e.g., the boundary of the head. Without a gradient, the RM would not be visible. Moreover, the steeper the gradient, the stronger the temporal signal. RM pixels typically extend 1-dimensionally (along an edge).
2. RF pixels can be in non-edge areas of the image. Moreover, the temperature changes due to ex/inhalation generate areas with a smooth circular gradient, caused by thermal diffusion. Consequently, they typically extend 2-dimensionally in the image.
3. *RM signals* can be in phase, or in anti-phase with the *RF signal*, depending on the direction of the motion and the temperature gradient. The RF always results in warming regions during exhalation and cooling regions during inhalation, whereas the RM, for example, could be visible at the edge between blanket and face resulting in warm pixels becoming colder during inhalation, or at the edge between the infant’s head and the sheet, resulting in colder pixels becoming warmer during inhalation.

### 2.2. Materials

#### 2.2.1. Experimental Setup

**Figure 1:**
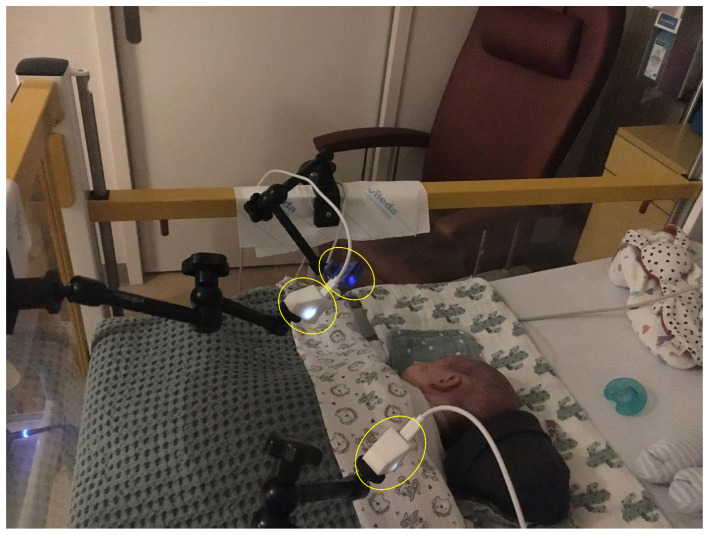
Experimental setup. Reprinted with permission from [32] © The Optical Society.

The videos were collected using three FLIR Lepton 2.5 cameras, these devices were chosen based on their relatively low cost, which allows the use of multiple cameras improving the coverage of the patient. The cameras are sensitive in the Long-Wave Infrared (LWIR) between 8 and 14μm, have a resolution of 60×80 pixels, and an average frame rate equal to 8.7 Hz. The three cameras were positioned around the infants’ open bed as visible in Figure 1. The video acquisition was performed using MATLAB (MATLAB 2018b, The MathWorks Inc., Natick, MA, USA). The CI from the patient monitor (Philips MX800) was acquired as the reference respiratory signal. An artifact was generated to allow the synchronization between the thermal videos and the patient monitor. For more information regarding the setup we refer to [32].

#### 2.2.2. Dataset

The videos were collected on infants who were nursed in an open bed in the Neonatal Medium Care Unit of the Máxima Medical Centre (MMC) in Veldhoven, The Netherlands. The study received a waiver from the ethical committee of the MMC and the infants’ parents signed an informed consent before the study. The thermal videos amount to a total of around 42 h acquired from 15 infants.

A manual annotation was performed by the first author to analyze the content of the videos. All events visible in the videos were annotated. In particular, the movements of the infants were divided into three main categories, still, type 1 motion, and type 2 motion. Still is when all body movements are absent apart from RM, type 1 and 2 motion were defined to differentiate between movements including the chest and movements involving only other parts of the body. Additionally, the presence of a soother was annotated, as this may affect the algorithm. Caregiver and parent interventions, baby out of bed, motion of the camera, and unsuitable camera view were also annotated. For more details on the annotation refer to [33].

For this study we focus solely on the video segments in which the infants are still, all other events were neglected. The total absence of body motion was preferred to allow an accurate annotation of the RF pixel location, detailed in Section 2.2.3. Moments in which the infant was still but had the soother were also not considered since the soother may end up physically hiding RF. The moments in which the infants were still for at least 30 s amount to 339 min considering all the 15 infants. Segments shorter than 30 s were not used.

**Figure 2:**
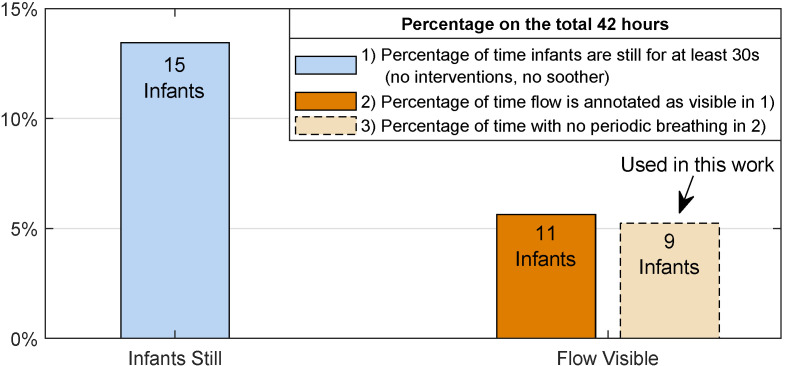
Percentages of video segments in which the infants are still with a minimum duration of 30 s, with flow visible, and with no periodic breathing. All percentages are reported with respect to the total recording time of 42 h.

To eliminate video fragments without visible flow, the first author performed an annotation by watching the unprocessed videos. The main strategy was looking for pseudo-periodic temperature variations at nostrils or textiles around the infant. Knowing that RF was present in the video segments was required for the development and testing of the algorithm. This selection results in around 142 min unequally split on 11 infants. Moreover, two babies presented periodic breathing, a benign breathing pattern made of an alternation of COBs and normal breathing [34]. These infants were removed from the usable data since the presence of COBs, not annotated, would cause underestimating the performance of the RF pixels selection and of the OA detection. Therefore, our dataset for this study amounts to around 132 min split in 87 segments and unevenly distributed on 9 infants. Figure 2 presents the distribution of the data and the percentage on the total 42 h. Table 1 shows the infants’ information, all the infants are in supine position except Infant 4 who is in prone position and Infant 1 is the only infant with a nasal cannulae. Moreover, Infant 2 and Infant 6 are the only infants in this dataset that do not have a nasogastric feeding tube. For further comments about the flow visibility in the recordings, refer to Section 4.

#### 2.2.3. Annotation of the Respiratory Flow Pixels Location

**Figure 3:**
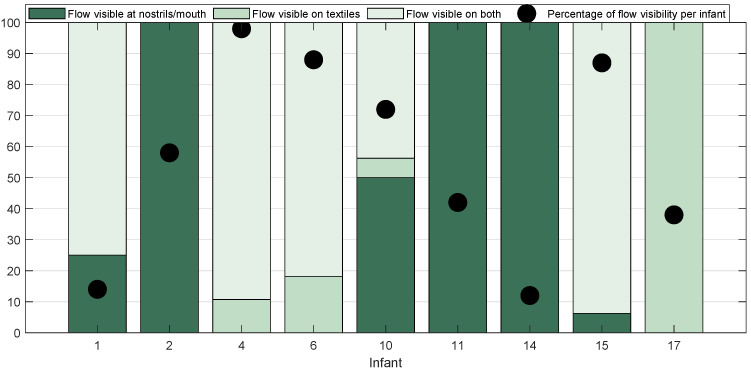
Information on the location of the annotated RF pixels for each infant and the percentage per infant of segments with flow visible compared to the segments annotated as still for at least 30 s.

To obtain a reference *RF signal* and to be able to estimate if our algorithm selects the correct pixels, each video segment of each infant was examined to locate the RF pixels. MATLAB was used for the manual annotation, and bounding boxes were used to annotate all the frame regions affected by airflow. This annotation was performed by the first author. Regions containing flow may occur at the nostrils, on the mouth, and/or on textiles surrounding the infants’ face, these were all considered to be valid RF pixels positions. The RF pixels were annotated at the location where pseudo-periodic temperature variations were visible in the thermal videos. Figure 3 shows the relative importance of the areas annotated as RF pixels in our videos. The figure also shows the individual percentage per infant of segments with flow visible compared to segments with the infant still for at least 30 s. The annotated RF pixels were used as the ground truth to evaluate the performance of the automatic RF pixel detection. A *Reference RF (RefRF) signal* was obtained by averaging together all the annotated RF pixels and was used for comparison purposes with the *RF signal* obtained using our algorithm.

#### 2.2.4. Obstructive Apnea Simulation

Our dataset did not include naturally occurring OAs, however, we wanted to include a proof of concept of enhanced OA detection with RF monitoring. Therefore, we built a dataset with simulated OAs, using the earlier videos. In total, we simulated 87 OAs. To simulate an OA, we substituted the annotated RF pixels with noise-pixels from the images for a time period of 10 s in the middle of each video segment. The low-frequency content of the noise-pixels was removed and replaced with the low-frequency content of the original RF pixels. In this way, all RF pixels will contain noise for the selected 10 s and the RM pixels are, instead, not altered.

### 2.3. Method

**Figure 4:**
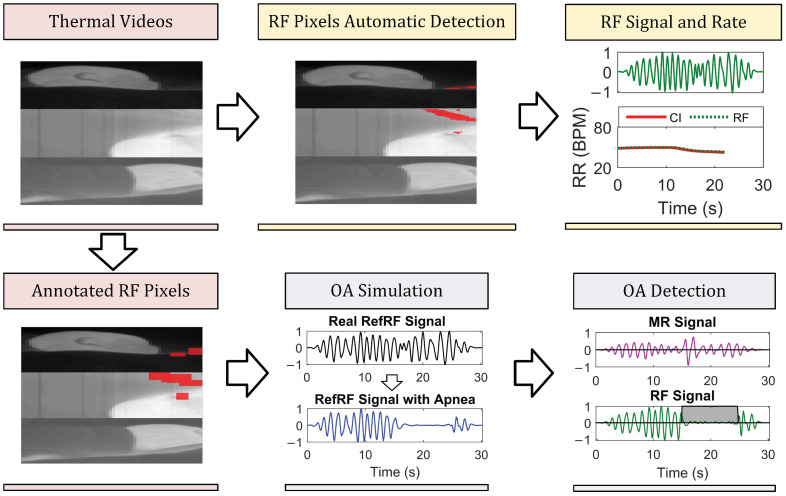
Summary of the processing and example results. RF pixels are automatically detected in the thermal videos and used to calculate the *RF signal* and the flow-based RR. Moreover, the RF pixel location is manually annotated. The annotated RF pixels are substituted with noise to simulate the occurrence of an OA. A COB-detector is used to compare the performance in OA detectability between the *RF signal* and the *MR signal*. The thermal frames shown are a vertical combination of three cameras visualizing the infant from different points of view.

The identification of the RF pixels is based on five features, partially already introduced in [33], combined with the new use of a bank of Gabor filters. These filters allow us to exploit the characteristic of RF pixels, i.e., that they typically occur in 2D-smooth areas. Using the chosen pixels, a *RF signal* and the flow-based Respiration Rate (RR) were obtained. Moreover, OAs were simulated in the 87 video segments and our previously proposed method for the detection of COBs [35] was used. This allows comparing the detectability of the OAs in the different respiration signals we obtain from the thermal videos (i.e., *MR signal* and *RF signal*). These steps are summarized in Figure 4.

#### 2.3.1. Preprocessing

The thermal images coming from the three camera views were merged on the same image plane as explained in [32] and as visible in Figure 4, obtaining a single video with resolution 180×80, i.e., M×L. Each pixel time domain signal was interpolated with a 1D linear interpolation to compensate for the uneven sampling rate. The resulting frame rate is 9 Hz, close to the average frame rate of the FLIR camera. This was also explained and used in our previous works [32,33].

#### 2.3.2. Respiratory Flow Detection

The method for the automatic detection of RF pixels, is based on the aspects explained in Section 2.1. Briefly, a set of 5 features is combined to identify the RF pixels. A flow-core-pixel, i.e., a pixel that is most likely to belong to the RF pixels, is selected as it will be used as a basis for the calculation of one of the features. Gabor filters are introduced for the accurate selection of the flow-core-pixel. The time domain signals of each pixel in each window are referred to as $x_{m,l}\left(nT_{s}\right)$, where (m,l) indicates the pixel position, and $n=0+\left(j-1\right)/T_{s},\;1+\left(j-1\right)/T_{s},\;\ldots ,\;\left(N-1\right)+\left(j-1\right)/T_{s}$. Each window is identified by the integer *j*, and consists of N=72 consecutive samples in an 8 s fragment, sliding in steps of 1 s. The sampling period $T_{s}$ equals 0.111 s.

Gabor filters are well-known bandpass filters used in image processing for texture and edge detection. The kernel is formed by a sinusoidal carrier and a 2D Gaussian envelope. Several Gabor filters can be generated by varying the spatial frequency of the sine wave and the orientation of the filter. By applying a set of filters to an image, edges and textures can be emphasized. Considering the properties of the distribution of RF pixels and RM pixels, we apply a bank of Gabor filters by varying the orientation and the spatial frequency aiming at locating RF pixels, which should have a similar response for all orientations. For RM pixels, on the other hand, we expect a higher response in specific directions, being mostly along, possibly curved, lines. We used the MATLAB built-in function to generate the bank of Gabor filters, i.e., *gabor*. We empirically selected a set of parameters for the orientation and for the spatial frequency of the filters, λ=3,4,…,8 pixels/cycle and $\theta =10^{\circ },\;20^{\circ },\;30^{\circ },\;\ldots ,\;170^{\circ }$. Multiple spatial frequencies were chosen to allow the method to work with both flow visible at nostrils/mouth or flow visible on textiles, as these usually produce regions affected by flow with different sizes. The bank is applied to an input map called *Flow Map*, which will be defined later, by convolving the input map with each Gabor filter. In particular:

(1) $$\Psi \left(\lambda ,\theta \right)=|\hat{\mathrm{FM}}⊗\Gamma \left(\lambda ,\theta \right)|,$$

where Γ(λ,θ) represent a Gabor filter, and $\hat{\mathrm{FM}}$ is the input map. The Ψ(λ,θ) are the magnitudes of the Gabor responses for each spatial frequency λ and orientation θ. We select the flow-core-pixel by multiplying all the Gabor responses Ψ(λ,θ). The flow-core-pixel is the pixel corresponding to the highest value in the image resulting from the multiplication:

(2) $$\left(m_{p_{f}},l_{p_{f}}\right)=\underset{\left(m,l\right)}{arg\;max}\left(\prod_{\lambda ,\theta }\Psi \left(\lambda ,\theta \right)\right).$$

Therefore, $\left(m_{p_{f}},l_{p_{f}}\right)$ indicates the position of the flow-core-pixel in each window. The map given as input to the Gabor filters is called *Flow Map* and is a combination of 5 features. In particular:

(3) $$\mathrm{FM}=\hat{C}\cdot {\tilde{C}}_{flow}\cdot \tilde{Q}\cdot \tilde{W}\cdot \left(J-G\right).$$

${\tilde{C}}_{flow}$ is a new feature introduced to locate RF pixels more accurately, and called *Covariance Map*. Each element represents the covariance between the signal of the flow-core-pixel found in the previous window and the signal of the other pixels in the video segment. ${\tilde{C}}_{flow}$ is the normalized version of the *Covariance Map*:

(4) $$c_{flow_{m,l}}=\left\{\begin{array}{cc} 1 & \mathrm{if}\;j=1\\ \frac{1}{N}\sum_{t=1}^{N}{\hat{x}}_{{\left(m_{p_{f}},l_{p_{f}}\right)}_{j-1}}\left(t\right)\cdot {\hat{x}}_{m,l}\left(t\right) & \mathrm{otherwise}. \end{array}\right.$$

$c_{flow_{m,l}}$ represents, therefore, the covariance between the signal of the chosen flow-core-pixel in the previous window ${\left(m_{p_{f}},l_{p_{f}}\right)}_{j-1}$ and the signal of a pixel in position (m,l), ${\hat{x}}_{{\left(m_{p_{f}},l_{p_{f}}\right)}_{j-1}}\left(t\right)$ and ${\hat{x}}_{m,l}\left(t\right)$ are the filtered time domain signals, while *t* is an index that sweeps through the samples in the *j*th window. The time domain signals were filtered with a passband between 30 and 110 Breaths Per Minute (BPM), i.e., the expected breathing frequency range of an infant. The $c_{flow_{m,l}}$ were then normalized resulting in a matrix between −1 and 1, i.e., ${\tilde{C}}_{Flow}$. The covariance was preferred to the correlation coefficient because it allows taking into consideration also the standard deviation of the time signals, which is advantageous assuming the biggest thermal variations are associated with respiration. Moreover, the sign of the covariance was kept which allows rejecting anti-phase signals, which can only originate from motion.

The other 4 features in Equation (3) were previously developed to obtain a *MR signal* from thermal videos, for a detailed explanation refer to [33]. These features were designed to locate MR pixels but can be adapted for the identification of the RF pixels. Q is called *Pseudo-periodicity* and is based on the estimation of the height of the normalized spectrum’s peak. W is called *RR Clusters* and is based on the application of a 2D non-linear filter for the detection of pixels that have similar frequencies nearby. G is *Gradient*, which identifies the edges of the thermal images. These three features were used to identify a core-pixel, i.e., a pixel that best represents the *MR signal*. Once a core-pixel is found the Pearson correlation coefficient is used to locate all the other pixels containing respiration signals and the *MR signal* is obtained by averaging these pixels together as explained in our previous work [33]. The Pearson correlation coefficients obtained between the core-pixel and all the other pixels are arranged in a matrix called *Correlation Map* and indicated with C. The *Correlation Map* obtained from the core-pixel can be used to locate the MR pixels. We binarized this map by applying an empirical threshold $\xi_{1}$ on the absolute values equal to 0.6:

(5) $$\hat{C}=\left|C\right|>\xi_{1}.$$

The *Flow Map* can be obtained by combining this binarized *Correlation Map* with the *Covariance Map* and the other features as explained in Equation (3). $\tilde{Q}$, $\tilde{W}$, and G represent the *Pseudo-periodicity*, *RR Clusters*, and *Gradient* features respectively, the tilde is used to indicate that the features were normalized between 0 and 1, G is already binary. J is an M×L matrix containing all ones and therefore the combination with the *Gradient* feature gives a weight equal to 1 to the non-edge regions. The *Flow Map* was then binarized by applying an empirical threshold, $\xi_{2}$ equal to 0.2:

(6) $$\hat{\mathrm{FM}}=\mathrm{FM}>\xi_{2}.$$

Even though the combination of these features allowed removing most of the RM pixels from the selectable pixels, in the first window the *Covariance Map* is not computed and some of these pixels may still be present in the binarized *Flow Map*. Moreover, considering the flow-core-pixel is used to estimate the *Covariance Map* in the following windows, the detection of the right pixel is particularly important. Additionally, the *Flow Map* may still contain some noise-pixels as well as the flow ones. Therefore, to select the flow-core-pixel accurately, we introduced the bank of Gabor filters, and the $\hat{\mathrm{FM}}$ was given as input to the bank, as done in Equation (1).

The RF pixels are therefore detected, in the first window of each video segment only the flow-core-pixel is used, afterwards, all non-zero pixels in $\hat{\mathrm{FM}}$ are considered RF pixels:

(7) $$P_{flow}=\left\{\begin{array}{cc} \left(m_{p_{f}},l_{p_{f}}\right) & \mathrm{if}\;j=1\\ \left(m,l\right)\;:\hat{\mathrm{FM}}\left(m,l\right)=1 & \mathrm{otherwise}. \end{array}\right.$$

**Figure 5:**
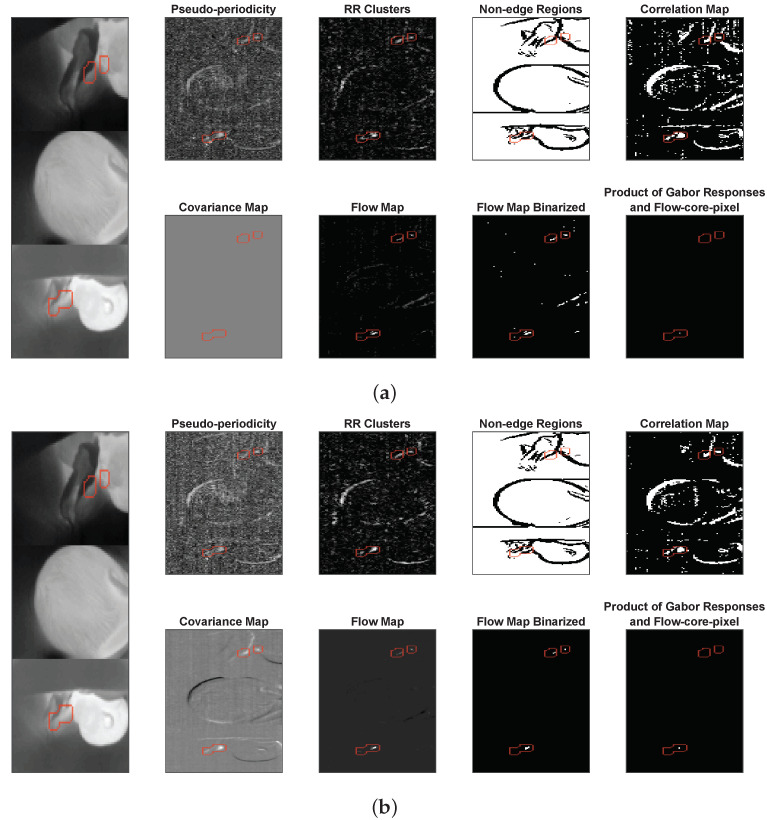
Example of features used for the detection of the RF pixels, the location of the annotated RF pixels is indicated with a red perimeter: (**a**) the features and the choice of the first flow-core-pixel; (**b**) the features used to locate the RF pixels in the next window.

$P_{flow}$ is, therefore, a set containing the positions of the detected RF pixels. The conditions for RF pixel detection are quite strict, it could happen that no pixel is found, in that case the previously chosen RF pixels are used in the current window as well. The *RF signal* is obtained by averaging together all RF pixels contained in $P_{flow}$. An example of the features in the first window is shown in Figure 5a, and the features obtained in the following window in Figure 5b. The figures show the advantage introduced using the *Covariance Map*, rejecting anti-phase RM pixels. As a consequence the *Flow Map* in Figure 5b does not contain RM pixels compared to the *Flow Map* in Figure 5a.

The *MR signal* is also obtained from the videos, using our method previously described in [33], and will be used for comparison purposes.

#### 2.3.3. Obstructive Apnea Detection

We adjusted our previously published COB-detector [35] to evaluate the detectability of OAs, which were simulated as indicated in Section 2.2.4. The COB-detector assumes that COBs can be detected by monitoring sudden amplitude changes and it is based on the comparison of a *short-term standard deviation* and a *long-term standard deviation*. The only adaptations applied to our previous published implementation concern the length of the windows for the calculation of the two standard deviations. The duration of these windows was chosen in [35] based on the targeted COB. In particular, the window for the calculation of the *short-term standard deviation* should be close to the minimum COB duration, an apnea of 10 s. Although, the window for the *long-term standard deviation*, which is calculated as median of the *short-term standard deviations*, must be higher than the COB duration. Otherwise, the *long-term standard deviation* will dynamically adapt to the standard deviation during the apnea event (i.e., detecting the cessation of the event while the apnea is still ongoing). In our current implementation, the *short-term standard deviation* is calculated using 8 s windows, which is the same sliding window approach used for the RR estimation. The *long-term standard deviation* is calculated in a window of 15 s. This window could be reduced to 11 s considering the fact that this is closer to the designed duration of the OA, but we kept it higher to easily adapt to non-simulated cases.

The *RF signal* and *MR signal* were obtained as described in the previous Section using our dataset with simulated OAs described in Section 2.2.4. The COB-detector was applied to the *RefRF signal*, as reference of the results achievable when monitoring RF, on the *RF signal* obtained from applying our method, and on the *MR signal* to highlight the limitations of monitoring this type of signal when aiming at apnea detection.

#### 2.3.4. Evaluation Metrics

To evaluate the performance of the RF pixel detector, we used the annotated RF pixels as a reference. In each window, we evaluate the percentage of detected RF pixels that belong to the annotated RF pixel areas. In particular:

(8) $$PF\left(j\right)=\frac{\#(P_{flow}⋂P_{ann})}{\#(P_{flow})}\cdot 100,$$

the symbol # is used to indicate the cardinality of the sets, and $P_{ann}$ is a set containing the annotated RF pixels. Moreover, to estimate the number of RM pixels erroneously included in the $P_{flow}$ set, we calculate the percentage of the detected RF pixels that belong to the pixels used to calculate the *MR signal* after removing $P_{ann}$ from the set. Formally:

(9) $$PM\left(j\right)=\frac{\#(P_{flow}⋂\;\left(P_{m}-P_{ann}\right))}{\#(P_{flow})}\cdot 100,$$

with $P_{m}$ indicating the set of pixels used to obtain the *MR signal*. The PF(j) and PM(j) are then averaged to obtain an average percentage of correct and incorrect pixels detected in each video segment. The Mean Absolute Error (MAE) is also estimated to compare RRs obtained using the *RF signal*, the *MR signal*, or the *RefRF signal* and the RR of the CI reference.

For the OA detection step, accuracy (ACC), sensitivity (SE), and specificity (SP) are calculated, by comparing the OA detection result with a template signal. The template signal has been built to be equal to one in the segment containing a simulated OA, and to zero in the rest of the signal. ACC, SE, and SP are calculated as defined in [35,36], i.e., by considering the total duration of the time intervals with OAs correctly and incorrectly detected (time true positive and time false positive), and correctly and incorrectly not detected (time true negative and time false negative).

## 3. Results

**Figure 6:**
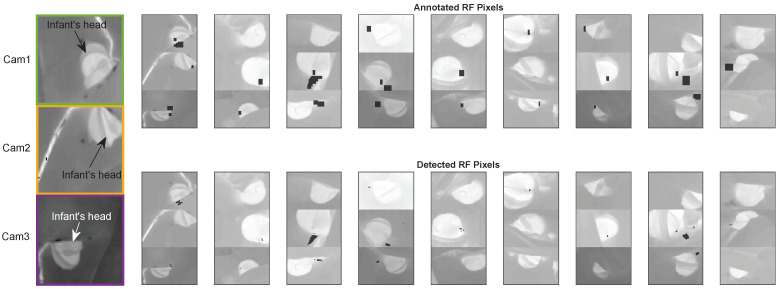
An example thermal image with a description of the content, and examples of the annotated RF pixels and the detected ones for the different infants included.

Figure 6 shows an example for each infant of the detected and the annotated RF pixels. This figure clearly shows the variability in flow location and infant positions contained in our relatively small dataset. Please note that each video segment of each infant may have a different flow location, due to the infant moving, therefore, this example figure does not cover all the cases present in the dataset.

**Figure 7:**
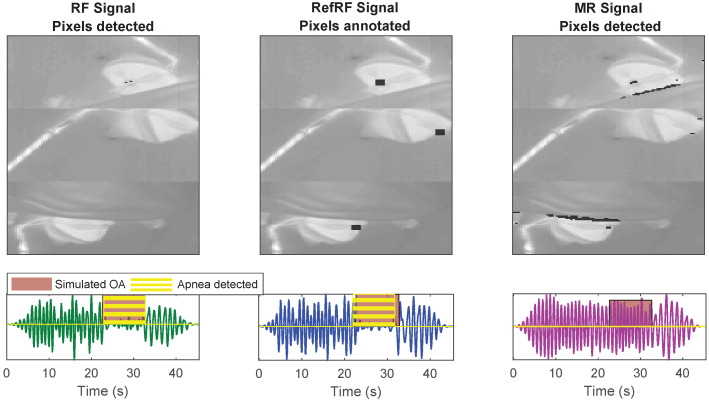
Example results of the automatic pixel detection step and of the OA simulation and detection step. The *RefRF signal* shows the simulated OA, the *RF signal* calculated using the automatically detected RF pixels is also able to detect the OA. In the *MR signal*, however, the OA is not visible, being the signal obtained by mixing RM and RF pixels.

The results of the RF pixel detection step and the MAE obtained for the different respiration signals obtained are shown in Table 2. The percentage of correct RF pixel detection, PF, is on average equal to 84.28%, and PM, RM pixels erroneously included, is on average 0.35%. The average MAEs obtained by comparing the RRs of the CI reference with the one of *RF signal*, *RefRF signal*, and *MR signal* are respectively 2.20 BPM, 1.85 BPM, and 2.11 BPM. Moreover, Table 3 contains the results of the OA simulation and detection step, showing ACC, SE, and SP calculated using the three different signals obtained from the videos. The superior performance of the *RF signal*, average SE 73%, compared to the *MR signal*, average SE 23%, in detecting the simulated OA is clearly shown here. Therefore, by monitoring the *RF signal* instead of the *MR signal* there was a gain in SE of around 50%, the SP also improved. An example of the OA simulation and the results of the COB-detector are visible in Figure 7, the images also show the pixels used to obtain the different signals.

## 4. Discussion

The proposed method obtained promising results in the automatic identification of RF pixels in thermal videos, obtaining *RF signals*. Based on our annotation, the time in which the RF was visible in the video segments and where the infants were still, amounts to around 142 min split between 11 infants. Therefore, for 4 infants, flow was never visible in the recordings due to the reasons we shall discuss here. The average percentage of still segments with a minimum duration of 30 s that were annotated to have flow visible for the 11 infants is 49% with a maximum of 98% reached by Infant 4.

Different aspects can affect flow visibility in thermal videos. First, the relative position between the infant face and the camera plays an important role in the visibility of RF at the nostrils, and since infants move, the cameras’ positions were not always optimal in our study. In addition, the blanket may end up covering the infant’s nose/mouth where flow is expected to be visible. The flow visibility annotation was performed by visual inspection of the unprocessed videos, it is possible that flow was present in some recordings but not directly visible due to a low contrast and therefore, neglected in this work. We cannot draw conclusions on whether the low-resolution of our setup had an influence on the flow visibility or on whether the thermal sensitivity of the cameras was insufficient as well. Moreover, other possible factors which make flow detection more complex in infants compared to adults were also mentioned by Abbas et al. in [20], such as the reduced lung volume or the small nasal aperture.

Solutions should aim at maximizing RF visibility in thermal videos. An array of cameras could be used to ensure the visibility of the nostrils area in the videos. Moreover, most of the infants in our dataset are in supine position, which is recommended for infants with a higher postnatal age. However, in NICUs most of the infants in the incubators are in prone position. We expect the prone position to increase the flow visibility on the textiles surrounding the face. Infant 4 was the only infant in prone position in our study and this infant has the highest percentage of flow visibility when the infant is still, as visible in Figure 3. Some of the other infants, such as 15 or 6, have also flow visible on the textiles even if they are positioned supine, thanks to the head position.

Our automatic RF pixel detection resulted in a percentage of correct RF pixels detected, PF, of 84% as shown in Table 2. Based on PM, RM pixels were hardly mixed in, which indicates that the edge removal strategy left a very limited number of RM pixels in the selectable pixels. It should be noted that PM is an estimation of the RM pixels erroneously included, as the RM pixels used for the calculation are the detected MR pixels after removing the annotated RF pixels. This is, therefore, dependent on the MR pixels detected by our previous algorithm, which are selected based on a threshold on the correlation with a core-pixel [33]. PM may be therefore underestimated, an annotation of the RM pixels may be needed to accurately estimate PM, the remaining percentage of pixels would then belong to the noise-pixels category. The PF was relatively low for Infant 10, 41%. In some of the recordings with the flow visible at the nostrils, due to a combination of camera position, head position, and temperature gradient between the nose and face, the RF pixels were not correctly identified. Therefore, the removal of the edge caused problems if all RF pixels were located on an edge, which can occur if the flow is visible only at the nostrils and if the relative position between camera and infant’s face causes the nose to be at the edge of the face. Although this last problem could be overcome with a different camera position, further complications are the nose having a lower temperature compared to the face creating additional edges in the image, and the presence of a nasogastric tube which can also create gradients close to the nostrils. The MAE for the *RF signal* was slightly higher than the MAE obtained with a *MR signal*. Compared to the *RefRF signal* MAE, the one for the *RF signal* was significantly higher for infants 4 and 10. The second one is linked to the wrong pixel selection, and similarly, in the case of Infant 4, in some of the segment noise-pixels were selected instead of RF pixels causing a large MAE. In this case, the flow was also visible on the textiles, however, due to the infant position, the removal of the edges caused the flow region on the textiles to have a more linear shape, the flow was therefore not selected. These limitations are further corroborating the need for more cameras and views, which will allow visualizing RF, but also visualizing it away from the image’s edges. Our experiments prove that low-cost cameras provide a feasible solution, so adding more cameras should not lead to prohibitive costs, but workflow disturbances should be also considered and minimized.

The proof of concept for the OA detection indicates that there is indeed an advantage in monitoring the *RF signal* compared to the *MR signal*, the sensitivity drastically increased from 23% to 73%, and the specificity improved as visible in Table 3. This result was expected, since the RM pixels were left untouched for the OA simulation. However, if the main source of respiration in the video segments is RF, as is the case in Infant 6, then also the *MR signal* obtained a relatively good sensitivity in the OA detection. The lowest sensitivities for the *RF signal* are linked to the incorrect detection of the RF pixels, indeed infants 10 and 4 resulted in lower sensitivities. This is a proof of concept, and this method should be tested on thermal recordings of real apneas. MA is the most common apnea in infants, characterized by the presence of obstructed inspiratory effort segments as well as central pause segments. Leaving the RM pixels unaltered for the OA simulation is a simplification, as the inspiratory effort of MAs and OAs can present changes in the amplitude and/or frequency compared to the *RM signal* pre-apnea [34].

Moreover, an important discussion point is the presence of an ambiguity between the absence of RF, leading to a possible apnea alarm, and the absence of RF visibility. This ambiguity can again be mitigated by increasing the number of camera views, maximizing RF visibility. However, for infants covered with a blanket this may not be a solution as the blanket could hide the RF. Still, AOP resolves with maturation and, thus, occurs more often in an infant population that is commonly positioned in incubators, i.e., infants with low gestational and postmenstrual age [3]. Infants nursed in incubators are usually not covered by traditional blankets and, thus, the problem is less relevant in this situation. Given an optimal number of views, the ambiguity could still be significant after motion events, which were not included in this study. The method may be combined with a gross motion detector, similar to [33], and our algorithm for RF pixel detection would need to be reinitialized after a movement of the infant (because of the time dependency introduced in the calculation of the *Covariance Map*). The ambiguity would exist, then, between an apnea occurring after a movement and a position change due to the movement that hides RF completely. It would be quite complex differentiating between these two events. Indeed, apneas can be preceded by motor activity [37]; however, infants are unlikely to completely change posture on their own to hide RF from all camera views. If the posture is changed by caregivers, then a protocol could be introduced to make sure the cameras are in a good position to still visualize RF.

Finally, infants in NICUs may need respiratory support to treat severe AOP or to treat other diagnoses. Although the nasal cannulae may not directly limit the visibility of RF, as in the case of Infant 1 or as shown in [38], the use of a larger interface (nasal mask) will inevitably hide RF at the nostrils. If RF is not visible in the thermal videos due to one of the discussed reasons, RM would anyway be visible in most of the recordings [33], implying that the information available for apnea identification would be similar to the current one, i.e., CI. It should be specified that bradycardia and desaturation need to be also detected in the case of a COB of 10 s to define it an apnea, therefore, for a future complete unobtrusive monitoring of apneas in the NICU these vitals (heartbeat and oxygen saturation) need to be included as well [9].

RM information could be obtained from other technologies such as RGB/NIR cameras, cameras with depth sensing, pressure-sensitive mattresses, or radars. However, while the use of additional non-thermal cameras to monitor RM may result in a higher RR accuracy, it would also share limitations with the current thermal setup, e.g., absence of RM information when the blanket is positioned such as to cover the chest motion [32]. Therefore, as already suggested in [33] radars or pressure-sensitive mattresses could be used as complementary technologies to increase the accuracy of RM detection while simultaneously overcoming the blanket problem. Finally, the method was implemented and tested using MATLAB, and the videos processed offline. Further development would be required before moving to a possible real-time application in the clinic, e.g., embedded solution and algorithm optimization.

## 5. Conclusions

The method proposed in this publication was able to detect automatically RF pixels in thermal videos reaching an average percentage of correct pixels estimation of 84%. RM pixels were correctly rejected, and they were hardly erroneously selected, 0.35%. The MAE was slightly higher on average compared to the one of the *RefRF signal*, 2.20 and 1.85 BPM, respectively. The proof of concept for the OA detection indicates a clear advantage in monitoring an *RF signal* compared to a *MR signal*, the sensitivity increased from 23% to 73%. However, the method should be tested in thermal recordings containing real apneas. RF was annotated to be visible on average in the 49% of the segments in which the infant was still. The number of cameras and their position play an important role in RF visibility in thermal videos and require further analysis.

## Figures and Tables

**Table 1:** Information on the infants and the data used in this work.

Infant	Gestational Age	Postnatal Age	Total Duration	Number of
	(Weeks + Days)	(Days)	(Minutes)	Video Segments
1	26 w 4 d	59	4.84	4
2	38 w 5 d	3	1.86	3
4	26 w 3 d	59	26.93	28
6	40 w 1 d	6	17.53	11
10	26 w 4 d	77	24.48	16
11	26 w 4 d	77	8.29	4
14	32 w 2 d	11	3.62	3
15	35 w 1 d	8	33.84	16
17	27 w 5 d	16	10.78	2

**Table 2:** Percentage of correct RF pixel detection, PF, and RM pixels erroneously included, PM. MAE comparing the RRs of the CI with the RRs of the *RF signal*, the *RefRF signal*, and the *MR signal*.

Infant	PF	PM	MAE (BPM)		
			RF	RefRF	MR
1	99.67%	0.00%	0.68	0.67	0.64
2	86.30%	0.15%	1.27	1.26	1.42
4	80.99%	0.35%	3.62	2.51	2.16
6	95.68%	0.13%	0.74	0.71	1.26
10	40.93%	1.21%	4.83	2.98	2.71
11	78.93%	1.19%	1.64	1.30	1.56
14	91.57%	0.00%	2.91	3.14	3.56
15	84.65%	0.12%	2.13	2.00	4.02
17	99.84%	0.00%	2.00	2.11	1.62
**Average**	84.28%	0.35%	2.20	1.85	2.11

**Table 3:** Accuracy, sensitivity, and specificity of the OA detection for the *RF signal*, the *RefRF signal*, and the *MR signal*.

Infant	RF			RefRF			MR		
	ACC	SE	SP	ACC	SE	SP	ACC	SE	SP
1	95.49	82.92	96.36	97.74	95.38	98.33	86.09	0.00	100.00
2	88.28	66.78	98.09	97.12	96.76	97.51	76.46	23.83	97.83
4	91.25	61.61	97.32	97.02	95.85	97.15	76.82	31.05	86.42
6	95.42	94.04	96.07	98.43	99.18	98.23	87.09	79.31	87.10
10	89.21	15.96	98.45	97.69	95.68	97.94	82.98	5.73	92.37
11	95.53	74.09	99.86	99.86	95.70	99.65	87.31	6.42	93.09
14	98.16	86.81	100.00	98.31	89.51	99.83	84.11	18.64	94.71
15	95.93	80.82	98.31	98.88	96.41	99.18	76.83	22.37	81.53
17	99.85	97.52	99.89	99.86	96.43	99.98	91.12	22.46	92.50
**Average**	94.35	73.39	98.26	98.32	95.66	98.64	83.20	23.31	91.73

## Data Availability

The data are not publicly available due to privacy reasons.
